# Metabolic Syndrome-Related Kidney Injury: A Review and Update

**DOI:** 10.3389/fendo.2022.904001

**Published:** 2022-06-23

**Authors:** Lirong Lin, Wei Tan, Xianfeng Pan, En Tian, Zhifeng Wu, Jurong Yang

**Affiliations:** ^1^ Department of Nephrology, The Third Affiliated Hospital of Chongqing Medical University (Gener Hospital), Chongqing, China; ^2^ Department of Nephrology, Chongqing Kaizhou District People’s Hospital of Chongqing, Chongqing, China

**Keywords:** metabolic syndrome, chronic kidney disease, diagnosis, pathological characteristics, therapeutics

## Abstract

Metabolic syndrome (MetS) includes visceral obesity, hyperglycemia, dyslipidemia, and hypertension. The prevalence of MetS is 20-25%, which is an important risk factor for chronic kidney disease (CKD). MetS causes effects on renal pathophysiology, including glomerular hyperfiltration, RAAS, microalbuminuria, profibrotic factors and podocyte injury. This review compares several criteria of MetS and analyzes their differences. MetS and the pathogenesis of CKD includes insulin resistance, obesity, dyslipidemia, inflammation, oxidative stress, and endothelial dysfunction. The intervention of MetS-related renal damage is the focus of this article and includes controlling body weight, hypertension, hyperglycemia, and hyperlipidemia, requiring all components to meet the criteria. In addition, interventions such as endoplasmic reticulum stress, oxidative stress, gut microbiota, body metabolism, appetite inhibition, podocyte apoptosis, and mesenchymal stem cells are reviewed.

## 1 Introduction

MetS called “Syndrome X” at first, it refers to a pathological state of metabolic disorders of proteins, fats, carbohydrates, and other substances in humans. It is including hypertension, hyperlipidemia, hyperuricemia, hyperglycemia, central obesity and insulin resistance. MetS is a common risk factor for the morbidity and mortality of cardiovascular events such as myocardial infarction, stroke, sudden cardiac death and thrombosis (1–6), it is also an important cause of new-onset CKD and progression of CKD. With the increasing prevalence of MetS, its effects on the kidneys have attracted an increasing amount of attention from nephrologists. The prevalence of MetS and MetS-related renal injury are also different due to different races, lifestyles, and MetS diagnostic criteria. Therefore, we will summarize the epidemiology, pathogenesis, diagnosis and treatment progress of MetS-related renal injury.

## 2 MetS Definition and Prevalence

There is no uniform diagnostic standard for MetS in the world, and the World Health Organization (WHO, 1998) (7), National Cholesterol Education/Adult Treatment Panel III (NCEP-ATPIII,2001) (8), Modified NCEP-ATP III,2010 (9), American Heart Association (AHA, 2005) (10), International Diabetes Federation (IDF, 2005) (11), and the Chinese Diabetes Society (CDS,2020) (12) definitions are currently used. Components of the several diagnostic criteria include central adiposity, an impaired glucose tolerance, waist circumference (WC); blood pressure, high density lipoprotein, triglycerides (TG), and serum glucose. The WHO believes that the basic condition for diagnosing MetS is an abnormal glucose metabolism, diabetes, or insulin resistance (IR). The IDF believes central adiposity is a prerequisite for the diagnosis of MetS. Due to the different focus of several diagnostic criteria, the incidence of MetS in the same group is different.Therefore, to unify the diagnostic criteria of MetS, IDF, American Heart Association/National Heart, Lung, and Blood Institute (AHA/NHLBI), and other institutions published the MetS Joint Interim Statement (JIS) criterion (13). This standard no longer regards WC as a necessary condition for the diagnosis of MetS. The JIS criterion for defining MetS includes: (1) raised fasting blood glucose (FBG) or the use of hypoglycemic drugs; (2) increased blood pressure or the use of antihypertensive drugs; (3) increased plasma TG or the use of hypolipidemic drugs; (4) a decreased HDL-C; and (5) the Chinese criterion for central obesity (or visceral obesity) with a waist circumference of >85 cm for men and >80 cm for women. MetS was diagnosed if three or more of the above criteria were met (13). The diagnostic criteria of the China Diabetes Association (CDA) MetS of the Chinese Medical Association has a high similarity and coincidence rate with the JIS and NCEP-ATPIII (Kappa values are 0.730 and 0.774, respectively) (14). **Table 1** shows the common definitions of MetS.

**Table 1:** Criteria for diagnosing MetS.

	WHO,1998	IDF,2005	NCEP ATP III,2004	Modified NCEP ATP III,2010	AHA,2005	CDS,2020
	**Presence of impaired glucose tolerance with any 2 of the following criteria**	**Presence of central adiposity with 2 or more of the following criteria**	**Presence of 3 or more of the following criteria**			
**Serum glucose**	plasma glucose at 2h after glucose load ≥7.8 mmol/L	FPG ≥100 mg/dL (5.6 mmol/L) or previously diagnosed type 2 diabetes.	FPG≥110 mg/dL (6.1 mmol/L)	FPG≥100 mg/dL (5.6 mmol/L)	FPG ≥100 mg/dL (5.6 mmol/L)	FPG ≥6.1 mmol/L or plasma glucose at 2 h after glucose load ≥7.8 mmol/L
**WC**	–	M: > 90cm; F: > 80cm	M: >102 cm; F: >88 cm	M: >102 cm; F: >88cm(Asian origin, M: >90 cm and F: >80 cm)	M: >102 cm; F: >88 cm	M: ≥ 90cm; F: ≥ 85cm
**BMI**	>30 kg/m2					
**WHR**	M>0.90 ;F >0.85					
**Hyperrension**	≥140/≥90 mmHg	≥130/≥85 mmHg	≥130/≥85 mmHg	≥130/≥85 mmHg or current use of antihypertensive drugs	≥130/≥85 mmHg	≥130/≥85 mmHg
**HDL Cholesterol**	M: < 35 mg/dL (0.9 mmol/L); F: < 39 mg/dL (1 mmol/L)	M: < 40 mg/L (1.03 mmol/L); F: < 50 mg/L (1.29 mmol/L) or receiving treatment	M: <40 mg/dL (1.03 mmol/ L) ;F: <50 mg/dL (1.29 mmol/L)	M: <40 mg/dL (1.03 mmol/ L) ;F: <50 mg/dL (1.29 mmol/L)	M: <40 mg/dL (1.03 mmol/ L) ;F: <50 mg/dL (1.29mmol/L)	<40 mg/dL (1.04mmol/L)
**Triglycerides**	≥150 mg/dL (1.7 mmol/L)	≥150 mg/dL (1.7 mmol/L)or receiving treatment	≥150 mg/dL (1.7 mmol/L)	≥150 mg/dL (1.7 mmol/L)	≥150 mg/dL (1.7 mmol/L)	≥150 mg/dL (1.7 mmol/L)

It is estimated that the worldwide prevalence of MetS is 20 – 25% (15), but there are great differences in the prevalence of MetS in various countries. According to the literature, the lowest prevalence of MetS patients is only 6.1% and the highest is 55.6%. This may be due to different gender, age, racial, eating habits, education, medical security, nature of work, and living environment. From 1980 to 2012, the prevalence of MetS in the United States increased to 35% (16), however, the prevalence of MetS in the United States has been gradually decreasing in recent years. The data released by the National Health and Nutrition Examination (NHANES) in 2020 showed that the incidence rate of MetS was 24% in men and 22% in women (17). A meta-analysis from China showed that the prevalence of MetS was 24.5% between 2007 and 2015. By sex, the prevalence was 19.2% in men and 27.0% in females. The older the age, the higher the prevalence of MetS. The prevalence of MetS in aged ≥ 60 was 2.33 times higher than those in aged 15-39 (32.4% *vs.* 13.9%). The prevalence of MetS was 1.3 times higher in individuals living in urban areas than those living in rural areas (24.9% *vs.* 19.2%) (18). A joint survey study between the United States and China showed that the prevalence of MetS in the two countries was 36.6-37.3% and 23.0%, respectively. The highest prevalence of MetS is in aged 40-50 (19). MetS is also common in South Asian countries, including Afghanistan, Bangladesh, India, Maldives, Nepal, Pakistan, and Sri Lanka. The lowest prevalence of MetS was only 8.6%, and the highest was 46.1% (20). All were in South Asian, using the ATP III diagnostic criteria, Pakistani males had a higher prevalence of MetS (55.6% vs. 45.9%) (21), while Indian females had a 1.30-fold higher prevalence of MetS (50.9% vs. 39.2%) (22). From 2009 to 2013, the prevalence of MetS in Japan was 14.6% (648/4446). By sex, the prevalence was 20.6% in males and 6.1% in females (23). It can be seen that the prevalence of MetS is greatly affected by dietary habits, medical insurance, gender and age, especially postmenopausal women have a higher prevalence of MetS.

## 3 Association Between MetS and CKD

MetS and CKD are causal and influence each other. A large number of studies have confirmed that MetS can lead to changes in renal structure and function, such as a decreased glomerular filtration rate (GFR) and increased urinary microalbumin (24–27). A meta-analysis showed that the risk of CKD in MetS was 1.34 times higher than those without MetS (28). Another meta-analysis showed that MetS increased the risk of CKD by 50% (29). Many studies found that each component of MetS was associated with CKD. The more components there were, the higher the risk of CKD (odds ratio, 1.96; 95%: 1.71,2.34) (26, 29). However, a few reports have shown a nonsignificant association, which may have been related to the different diagnostic criteria used for MetS (30). Similarly, due to impaired renal function, microenvironment changes and disorder of glucose and lipid metabolism in patients with CKD, the incidence of MetS in patients with CKD is significantly higher than that in the general population. With the progress of CKD, the incidence of MetS gradually increases (31). A study in Thailand found that the prevalence of MetS in patients with CKD was 71.3%, and the prevalence of met in patients with ckd3a to 5 was 70.1%, 72.3%, 73.4% and 72.7% respectively, which was significantly higher than that in patients without CKD (32).

Recently, after using the “MetS score” and the “MetS factor” to refine MetS and its components, it was found that regardless of gender and racial, the higher the score of MetS, the higher prevalence of CKD, including GFR decline and microalbuminuria increase (33–35). When each component of MetS was evaluated separately, it was found that hypertension and increased LDL metabolism were associated with microalbuminuria and estimated glomerular filtration rate (eGFR), hyperglycemia and hypertriglyceridemia were associated with microalbuminuria (36). Sixty percent of the study population had MetS in CKD3-4. After more than 2 years of follow-up, CKD stage 3-4 patients with MetS had a 1.33 times increased risk of progression to ESRD. Among the components of MetS, hyperlipidemia and elevated blood pressure are more likely lead to the progression of CKD to ESRD; Low HDL cholesterol increases the risk of death; Impaired glucose metabolism is an important risk factor for ESRD and death (37).

## 4 The Pathogenesis of MetS-Related Renal Injury

**Figure 1:**
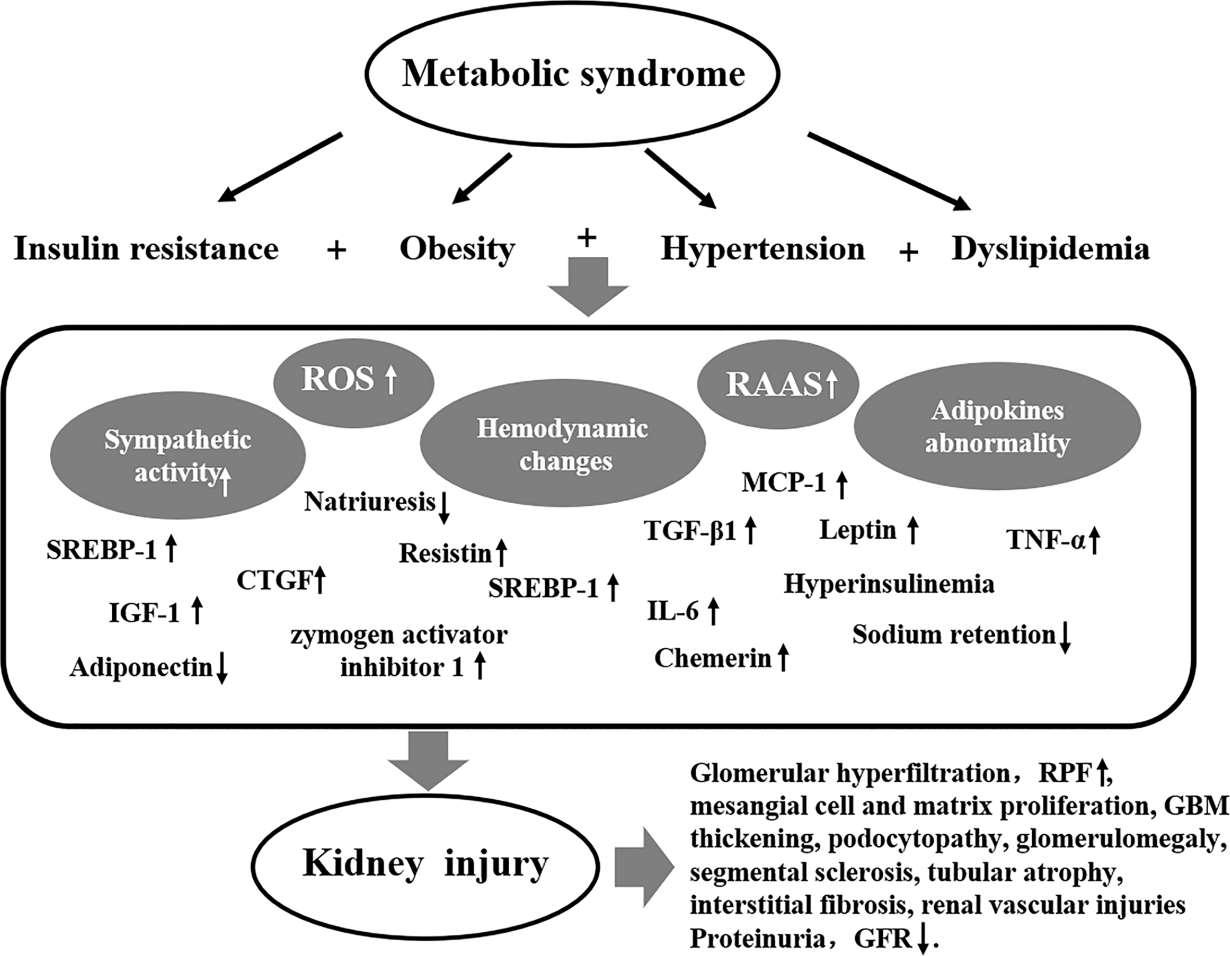
The pathogenesis of MetS-related kidney injury.

The pathogenesis of MetS-related renal damage is complex, including insulin resistance, obesity, hypertension, dyslipidemia, inflammation, oxidative stress, and endothelial dysfunction. The pathogenesis of MetS-related kidney injury is shown in **Figure 1**.

### 4.1 Insulin Resistance

Insulin resistance is essential factor of MetS, it is able to induce sodium retention and vascular endothelium vasoconstriction by antinatriuresis, which leads to RAAS activation and renal tubular lipid accumulation finally. This may be the important cause of MetS-related renal damage caused by insulin resistance (38, 39). Insulin resistance-related renal injury also includes transforming growth factor-β1 (TGF-β1), which is increased in the adipocytes of obese patients with insulin resistance and is responsible for the proliferation of mesangial cells and ultimately CKD, a potent initiator of disease (40–43). The sterol regulatory element binding protein-1 (SREBP-1) increases lipid droplet deposition in renal tubular cells and interstitial extracellular mechanisms (44–46), leading to tubular atrophy and interstitial fibrosis. Insulin like growth factor-1 (IGF-1) and dedifferentiation of vascular smooth muscle cells can induce connected tissue growth factor (CTGF), thus promoting renal tubular fibrosis Hyperglycemia in rat mesangial cells inhibits the degradation of the extracellular matrix by metalloproteinase 9, resulting in mesangial extracellular matrix proliferation and fibrosis (47–50). In addition, insulin resistance damages the microvessels of patients, especially the fundus, kidneys, muscles and cardiac arteries, and eventually damages the corresponding target organs (51).

### 4.2 Obesity

Hemodynamic changes, abnormal lipid metabolism, and dysregulations of the hormone response are the primary pathogeneses of obesity-Related Glomerulopathy (ORG) (52). A study found that aldosterone levels were significantly elevated in obese patients, hypertension and high WC were positively correlated with the level of aldosterone and negatively correlated with HDL (53). Activation of the RAAS and enhanced sympathetic activity in obese patients lead to increased levels of aldosterone, which reflexively lead to increased renal tubular reabsorption of sodium salts, resulting in water and sodium retention (54). The activation of RAAS in obese patients leads to hemodynamic changes such as increased of GFR and renal plasma flow (RPF), which causes glomerular hyperfiltration, compensated glomerulomegaly, segmental sclerosis, and promoting the progression of MetS-related renal damage (55–57). Hormone regulation disorder and ectopic lipid deposition in obese patients can directly or indirectly affect the structure and function of renal intrinsic cells (52). Hyperinsulinemia promotes the secretion of leptin by adipocytes, and leptin levels are significantly elevated in MetS patients (58, 59). Increased leptin secretion in MetS patients leads to kidney damage through the following two pathways, including (1) promoting the expression of TGF-β1 in renal parenchymal cells, increasing the production of type IV collagen, leading to tubular atrophy, interstitial fibrosis and glomerulosclerosis, (2) Leptin causes sodium reabsorption, leading to changes in renal hemodynamics (60, 61).

### 4.3 Hypertension

Hypertension is an important condition for the diagnosis of Mets. Hypertension in MetS patients is closely related to plasma aldosterone levels and sympathetic nerve activity (53). First, visceral adipocytes secrete a large number of substances such as angiotensinogen, which leads to the activation of RAAS (62–64). Second, the accumulation of excessive fat in and around the kidneys of patients with MetS compresses renal parenchymal cells, resulting in impaired pressure natriuresis (65–68). Thirdly, other hormone secretion disorders in MetS patients (including increased leptin, reduced adiponectin, etc.) lead to increased sympathetic nerve activity in the body (69–71), which are important reasons for hypertension in MetS patients. For a long time, the changes in renal structure and function caused by hypertension cannot be ignored, and it is also one of the important secondary causes of ESRD. Hypertension primarily causes kidney damage due to ischemia. Ischemia causes renal tubular, renal vascular, and glomerular damage, primarily renal tubular damage (72, 73). Ischemia can also increase the synthesis and secretion of angiotensin II (ANG II), which can further constrict blood vessels and lead to the proliferation of renal parenchymal cells, damaging the kidney through hemodynamic and non-hemodynamic effects (74).

### 4.4 Another Pathogenesis

During MetS, adiponectin, leptin, chemerin, resistin, IL-6, and tumor necrosis factor α(TNF-α) and other adipokines are abnormally secreted and released, or dysfunctional, which induces oxidative stress, endothelial dysfunction, inflammatory effects, and increased sympathetic activity, and finally lead to changes in renal function and structure (75–79). Furthermore, during Mets, adipose tissue secretion of pro-inflammatory factors increased, including macrophage chemoattractant protein-1 (MCP-1), macrophage migration inhibitory factor, chemokine ligand 5, and macrophage colony-stimulating factor. These pro-inflammatory factors can lead to inflammatory response, resulting in proteinuria and impaired renal function (80–82). The production of reactive oxygen species (ROS) in renal tissue increases due to inflammatory cell infiltration. ROS may cause damage to proximal tubules by interfering with renal tubular ion transport by altering renal and hemodynamics. ROS can also induce TGF-β1 and fibrinolysis through the activation of the nuclear factor-κ light chain enhancer and nicotinamide adenine dinucleotide phosphate oxidase (NADPH) pathways of activated B cells. The expression of pro-fibrotic molecules, such as zymogen activator inhibitor 1, thus aggravates the progress of renal fibrosis (83).IR is an independently risk factor in many diseases, it affects renal podocytes. Podocyte foot loss causes partial shedding of the glomerular filtration barrier (GFB), resulting in macromolecular leakage and proteinuria (84, 85). The adipose tissue secretes all components of the RAAS. During Mets, excessive activation of RAAS will lead to increased renal volume load and hyperfiltration, thus damaging the GFB, including endothelial cells, basement membrane, especially the podocytes (67, 86). In obese patients, a large number of lipid droplets can be found in renal innate cells, especially in podocytes. The deposition of lipid droplets leads to the depletion of renal cell energy, and ultimately the apoptosis of intrinsic renal cells, resulting in CKD and even ESRD (85, 87, 88).

## 5 Diagnosis of MetS-Related Renal Injury

MetS-related kidney disease is renal impairment in patients with MetS that includes glomerular hyperfiltration, eGFR < 60/mL/min per 1.73 m^2^, proteinuria and/or microalbuminuria, renal tubular dysfunction, ultrasound abnormalities (increased intra-renal resistive indices) (89), and histopathological abnormalities (90). Of course, MetS with CKD is not exactly MetS-related kidney disease. In addition, there is consistent evidence in the literature regarding the association between MetS and kidney stones (91, 92).

**Figure 2:**
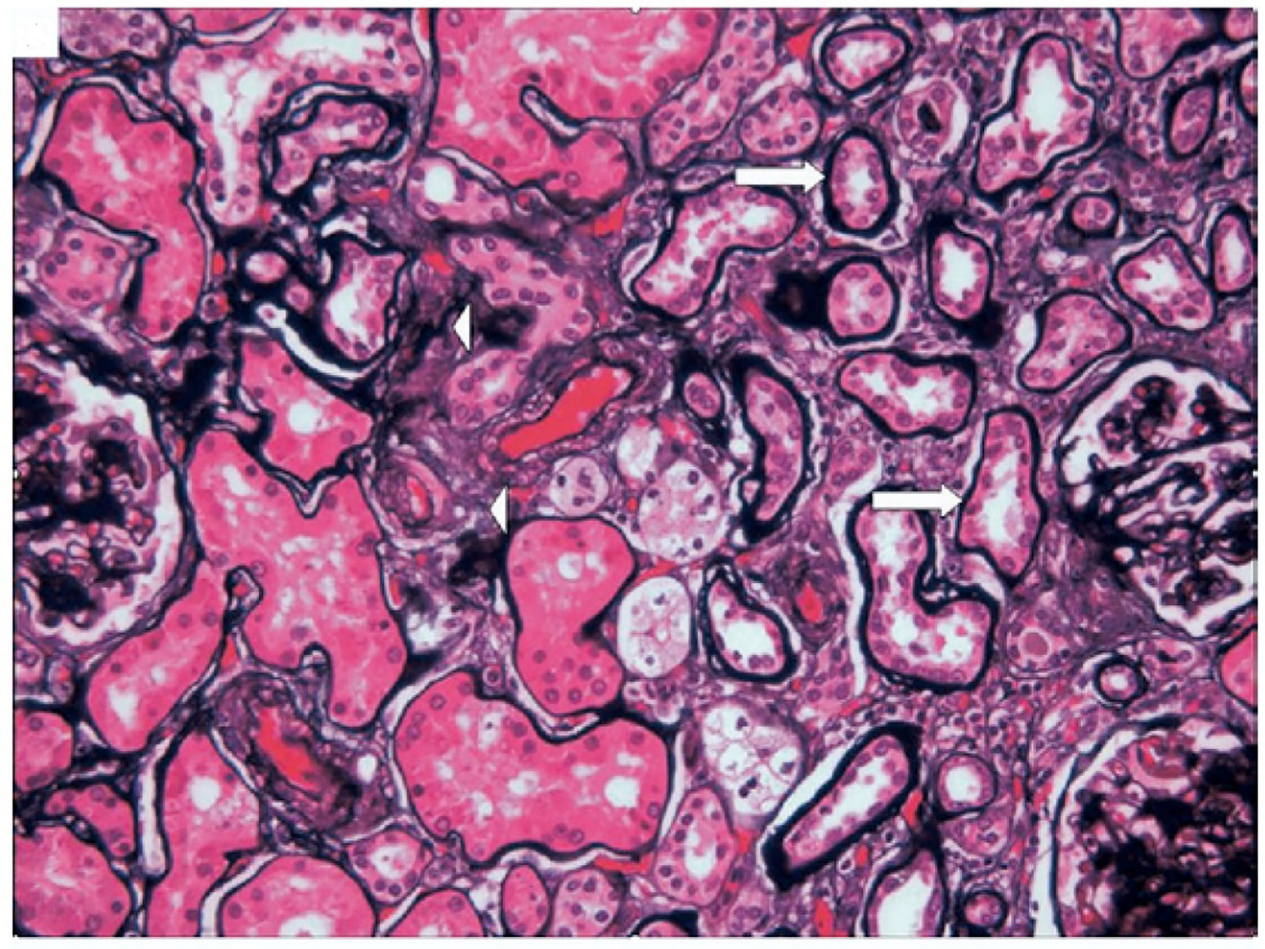
Kidney pathological characteristics for patients with MetS. Tubular atrophy (arrows) and interstitial fibrosis (arrowheads), PASM. This picture is from Mariam P et al., 2009 (98).

Several components of MetS may affect the kidney, or they may be combined to damage the kidney. Therefore, the damage of MetS to the renal tissue structure is also diverse. Kidney pathological characteristics in patients with MetS include glomerulomegaly, podocytopathy, mesangial cell and matrix proliferation, GBM thickening, global sclerosis, segmental sclerosis, tubular atrophy, interstitial fibrosis, and renal vascular injuries (arterial sclerosis and hyalinosis) (93–97). Kidney pathological characteristics for patients with MetS is shown in **Figure 2** (98).

Recent studies have found that some new biomarkers are significantly altered in the blood and urine of MetS patients, such as growth differentiation factor -11 (GDF-11). GDF- 11 is an important endocrine factor involved in the metabolic process of the body (99, 100), was found to be negatively correlated with body mass index and WC of MetS patients (101, 102). Growth differentiation factor 15 (GDF-15) is an endocrine factor involved in metabolism, and GDF-15 levels have been found to be significantly increased in elderly MetS patients and has been independently correlated with MetS (103, 104). A study from the Japanese found that urinary A-megalin is associated with the clustering number of MetS traits including hyperhomocysteinemia (105, 106). At present, Urinary podocyte-derived EVs (pEVs) is widely recognized as a specific biomarker for podocyte injury, and studies have found that it is significantly increased in secondary podocytes, such as early diabetic nephropathy, hypertensive nephropathy, and eclampsia (107–109). An animal study found that the urine pEVs of MetS model pigs increased significantly, Meanwhile, kidney histology confirmed the existence of podocyte and mitochondrial damage in MetS pigs. Therefore, urinary pEVs may be an early biomarker for MetS-related kidney injury (88). Whether the above indicators can be used as markers for the diagnosis of MetS-related nephropathy requires further study.

## 6 Management of MetS-Related Kidney Disease

A study found that patients with uncontrolled MetS had a 3.28 times higher risk of rapid decline in renal function than those who were controlled (110). Therefore, early treatment of MetS is beneficial to prevent and delay the progression of kidney disease. Specific measures include lifestyle change, managing weight control, hypertension, hyperlipidemia, and abnormal blood sugar. If each component of MetS is treated, each component can meet the standard. In recent years, studies have found that intervention targeting gut microbiota, oxidative stress, and inflammatory responses, and stem cell transplantation may help to alleviate Mets related nephropathy.

### 6.1 Lifestyle Interventions

Lifestyle interventions have always been important means of control MetS, including changing dietary patterns, focusing on a veggie–fruit–grains dietary pattern (111), adhering to aerobic exercise, a diet rich in medium-chain fatty acids and short-chain fatty acids, reasonable sleep, smoking cessation, and avoiding excessive intake of coffee. A study found that excessive coffee consumption (≥3 cups/day) increased the incidence of MetS by 1.5 times (112).

### 6.2 Weight Loss

Lifestyle interventions such as dietary restriction, change dietary Patterns, aerobic exercise, a diet rich in medium-chain fatty acids and short-chain fatty acids, reasonable sleep, and smoking cessation have always been important means of weight control. Studies have found that after dietary restriction or dietary restriction combined with aerobic exercise, serum creatinine and albuminuria decreased, GFR increased, renal hemodynamics improved, and the risk of kidney stone formation reduced with weight loss (81, 113–115). A study from Taiwan showed that compared with other dietary patterns, MetS patients with veggie–fruit–grains dietary pattern had better parameters of kidney function, including lower serum creatinine, blood urea nitrogen, and serum uric acid levels, and higher eGFR (111). A study of bariatric surgery in adolescents showed that eGFR increased by an average of 26ml/min/1.73m2 three years after surgery. Participants with albuminuria at baseline had improved significantly after operation (116). Similarly, after 6 years of follow-up for weight loss by gastric bypass, it was found that the patient’s metabolic-related indicators were well controlled, cardiovascular events were significantly reduced, and the risk of moderate to severe kidney disease was reduced by 45% (117). However, the existing literature demonstrated that despite its perioperative risks and short-term complications, surgical weight loss has a better long-term prognosis for cardiovascular and renal disease (116, 118–121).

### 6.3 Antihypertensive Therapy

Patients with hypertension should first adhere to a salt restriction diet. The second is the application of antihypertensive drugs. It is recommended to use angiotensin converting enzyme inhibitor (ACEI) and angiotensin receptor antagonist (ARB) to control hypertension in MetS patients, and this is beneficial to reduce Renin Angiotensin Aldosterone System(RAAS) activation, relieve glomerular hyperfiltration, and reduce proteinuria and hyperuricemia (122).RAAS blocker combined with exercise training can better reduce the hypertension, urine albumin-to-creatinine ratio, and serum creatinine of MetS patients (39, 123). Losartan can stably reduce blood pressure in MetS patients, while maintaining the normal circadian rhythm of blood pressure and achieve renal protection (124).

Animal studies have found that long-term administration of telmisartan can reduce the release of leptin from adipose tissue, thereby reducing proteinuria (125). Calcium channel blocker drugs, benidipine can also reduce the mean arterial pressure and resistance of the renal arterioles in MetS patients, and the urinary protein excretion rate is reduced by 1.5 times (126).

### 6.4 Lipids Adjustment

Statins have very solid evidence in controlling hyperlipidemia, stabilizing atherosclerosis, and reducing the risk of cardiovascular disease, and have become the first-line drugs for hyperlipidemia in MetS patients (127). Fibrates are also effective in hyperlipidemia, especially in the control of atherosclerotic dyslipidemia (128). In addition, the anti-inflammatory, antioxidant, antithrombotic, anti-fibrotic, and endothelial cell function improvement effects of statins can reduce proteinuria in patients with MetS and delay the progression of kidney disease (129). A study found that the benefit of cardiovascular protection in patients with MetS treated with statins was significantly higher than that without MetS. The eGFR in the MetS increased by 13.7mi/min/1.73m2, which was 2.36 times higher than that in MetS patients without statins (130). In MetS patients with CKD, it is safe to control hyperlipidemia with statins or fibrates. Is the combination of the two drugs safe for MetS with a variety of dyslipidemia? A study found that in MetS patients with CKD treated with low-dose statins combined with fibrate still have high safety and can significantly reduce proteinuria (131). Studies have found that the expression of 11beta-Hydroxysteroid dehydrogenase type 1 (11β-HSD1) is significantly increased in obesity and glucose and lipid metabolism disorders (132). 11β-HSD1 inhibitors can improve the lipid metabolism disorder in MetS (133–135).

### 6.5 Glucose Control

Metformin, pioglitazone, dipeptidyl peptidase-4 (DPP-4) inhibitors, glucagon-like peptide 1 (GLP-1) receptor agonists, and the sodium-glucose cotransporter 2 (SGLT2) inhibitor have been studied in patients with MetS. Metformin has good safety in the treatment of hyperglycemia and can enhance insulin sensitivity at the same time. It is the best solution for the treatment of MetS glucose metabolism disorder in children (136). Thiazolidinedione increases insulin sensitivity, improves IR and glycemic control, and also significantly reduces blood pressure, increases high-density lipoprotein (HDL), improves endothelial cell function and fibrinolytic activity, and reduces inflammation.

DPP4 inhibitors not only have definite hypoglycemic effect, but also play a protective role in cardiovascular, kidney and important target organs through the following mechanisms. Including regulating the levels of adenosine monophosphate-activated protein kinase and adiponectin levels in MetS mice, reducing inflammatory response and fatty liver (137), DPP-4/incretin axis reducing cardiovascular events (138), and improving hyperglycemia induced vascular lesions through endothelium-dependent Manner (139).

In recent years, GLP-1 receptor agonists have been used more and more widely. They not only exert the hypoglycemic effect, but also do not increase the risk of hypoglycemia. At the same time, they also have the effects of reducing weight, controlling hyperlipidemia, hypertension, and reducing inflammatory reaction, so as to prevent cardiovascular events (140). Liraglutide is a widely used GLP-1 receptor agonist, which has definite efficacy in reducing plaque formation and anti-atherosclerotic action (141).

Similarly, in addition to controlling hyperglycemia, SGLT2 also plays a role in reducing weight loss, controlling hypertension, increasing urinary sodium excretion and reducing edema by reducing sympathetic activity, improving insulin resistance (142), regulating renal sodium and urate transport and excretion in MetS patients (143, 144). It has a definite protective effect on cardiovascular and renal function in type 2 diabetes and MetS patients.

### 6.6 Chinese Herbal Medicines

In recent years, many Chinese herbal medicines and their extracts have achieved significant efficacy in the treatment of MetS and MetS- related kidney damage. They mainly improve components of the MetS and protect the kidneys by exerting anti-endoplasmic reticulum stress, antioxidant and anti-inflammatory activities.

Endoplasmic reticulum stress is an important pathogenesis of MetS kidney damage. Berberine can reduce urinary microalbumin, the body mass index, and postprandial blood glucose and triglyceride levels in MetS patients by regulating glucose and lipid metabolism, endoplasmic reticulum stress, inflammatory factors, insulin resistance, oxidative stress, and intestinal microbiota (145–148).

Osthol and Hibiscus sabdariffa L (HSL) have significant antioxidant effects. The mechanisms include inhibiting ketohexokinase activity and regulating adipogenesis; reducing oxidative stress by activating nuclear factor-erythroid 2-related factor (Nrf2) (149); promoting an increase in the enzymatic and non-enzymatic antioxidant systems, leading to a reduction in oxygen species (150). Ultimately, it promotes renal repair and delay renal progression in MetS patients.

Pycnogenol is an extract of pine bark, which is rich in bioactive substances such as procyanidins and catechins, has strong anti-inflammatory activities (151), affects endothelial function and reduces blood pressure (152). A randomized controlled study of ramipril alone and ramipril combined with pycnogenol in the treatment of MetS found that the combined treatment group decreased urinary albumin more significantly than the control group, and the renal cortical blood flow rate and renal function improved more significantly (123).

### 6.7 Other Treatments

#### 6.7.1 Probiotics

Gut microbiota can interfere with host metabolism, and the taxonomic species or abundance of gut microbes are affected by dietary patterns, lifestyles, and drugs. A study found changes in gut microbial taxonomic species in obese patients (153), and gut microbial-derived metabolites may induce subclinical inflammatory processes in MetS patients, leading to target organ damage (154).. Studies have found that probiotics have great benefits for obese and MetS patients by improving the body’s inflammatory state and reducing homocysteine and blood glucose levels (155). A randomized controlled study of patients with MetS found that the severity of MetS was significantly reduced after probiotic supplementation (156). Whether probiotics have a protective effect on MetS-related renal damage needs further study.

#### 6.7.2 Glycine

Glycine is a non-essential amino acid in the human body, but it participates in the important process of metabolism, as well as the process of immune regulation and anti-inflammatory. A study found that plasma the glycine level in MetS patients were lower than that in healthy. Glycine supplementation can improve a variety of clinical symptoms of MetS, such as abnormal glucose metabolism, overweight, hypertension, and hyperlipidemia (157). A study of 60 patients with MetS found that daily supplementation glycine (15 g/day) for 3 months, significantly reduced oxidative stress responses in the treatment group, including superoxide dismutase-specific activity and thiobarbituric acid reactive substances. The patient’s hypertension was significantly improved (158).

#### 6.7.3 Stem Cell Therapy

Mesenchymal stem cells (MSCs) can improve the various disorders of MetS such as abdominal obesity, hyperglycemia, hypertriglyceridemia, and hypertension. MSCs promote renal injury in diabetic mice (159, 160). A superior source of MSCs is from the umbilical cord -MSCs, where the cells can be obtained conveniently, less invasively, and have excellent regenerative and immunosuppressive properties (161). However, whether stem cell therapy can improve the renal tissue structure and function of MetS related renal damage has not been studied.

## 7 Discussion

With improvements in living standards and changes in lifestyle, the prevalence of MetS is increasing annually. In the future, MetS-related renal damage will become one of the key diseases for CKD prevention and treatment. We should focus on prevention and pay attention to early comprehensive intervention by including the control of body weight, hypertension, hyperglycemia, and hyperlipidemia, requiring all components to meet the criteria. Recently, studies have demonstrated that many new intervention measures, such as anti-inflammatories, antioxidants, the regulation of intestinal microorganisms, inhibiting appetite, stem cell transplantation, and other treatment methods, have achieved preliminary results and are expected to become new treatment targets for MetS related renal injury. This will reduce the prevalence of MetS renal damage and improve the prognosis.

## Author Contributions

LL, WT, xP, ET and ZW collected all the literature, carried out the analysis of data and outcome. LL mainly drafted the manuscript. JY revised and approved the final manuscript. Each author contributed important intellectual content during the drafting and revision of the manuscript. All the authors read and approved the final version of the manuscript to be published.

## Funding

This work was supported by the National Natural Science Foundation of China (No. 81770682), the Basic and Frontier Research Program of Chongqing (cstc2017jcyjBX0014) and the Chongqing Talent Program Project (cstc2021ycjh-bgzxm0090).

## Conflict of Interest

The authors declare that the research was conducted in the absence of any commercial or financial relationships that could be construed as a potential conflict of interest.

## Publisher’s Note

All claims expressed in this article are solely those of the authors and do not necessarily represent those of their affiliated organizations, or those of the publisher, the editors and the reviewers. Any product that may be evaluated in this article, or claim that may be made by its manufacturer, is not guaranteed or endorsed by the publisher.

## Abbreviations

MetS, Metabolic syndrome; RAAS, Renin Angiotensin Aldosterone System; GFR, glomerular filtration rate; eGFR, estimated glomerular filtration rate; CKD, chronic kidney disease; ESRD, end-stage renal disease; ACEI, angiotensin converting enzyme inhibitor; OS, oxygen species; Nrf2,erythroid 2-related factor; HSL, Hibiscus sabdariffa; HDL, high-density lipoprotein; DPP-4, dipeptidyl peptidase-4; SGLT2,sodium-glucose cotransporter 2; GLP-1,glucagon-like peptide 1; CVD, cardiovascular disease (CVD); ARB, angiotensin receptor antagonist; TMAO, trimethylamine N-oxide; LPS, lipopolysaccharide; MSCs, Mesenchymal stem cells; WHO, World Health Organization; NCEP-ATPIII, National Cholesterol Education/Adult Treatment Panel III; CDC, Chinese Diabetes Society; WC, waist circumference; TG, triglycerides; IR, insulin resistance; AHA/NHLBI, American Heart Association/National Heart, Lung, and Blood Institute; JIS, Joint Interim Statement; FBG, fasting blood glucose; CDA, China Diabetes Association; NHANES, National Health and Nutrition Examination; TGF-β1,transforming growth factor-β1; ORG,obesity-Related Glomerulopathy; RPF, renal plasma flow; ANG II, angiotensin II; TNF-α, tumor necrosis factor α; MCP-1, macrophage chemoattractant protein-1; ROS, reactive oxygen species; NADPH, nicotinamide adenine dinucleotide phosphate oxidase. GFB, glomerular filtration barrier; GDF-11, growth differentiation factor- 11; GDF-15, Growth differentiation factor 15; pEVs, podocyte-derived EVs; 11β-HSD11,11beta-Hydroxysteroid dehydrogenase type 1; MSCs, Mesenchymal stem cells.
